# Lifestyle school-based intervention to increase the proportion of adolescents free of components of the metabolic syndrome in an andean region of Peru

**DOI:** 10.17843/rpmesp.2022.391.9986

**Published:** 2022-03-31

**Authors:** Segunda Aydeé García Flores, Juana Aurelia Ninatanta-Ortiz, Martha Vicenta Abanto Villar, Katia Maribel Pérez Cieza, Rosa Ricardina Chávez Farro, Sara Elizabeth Palacios Sánchez, Franco Ronald Romaní Romaní

**Affiliations:** 1 Escuela Académico Profesional de Enfermería, Facultad Ciencias de la Salud, Universidad Nacional de Cajamarca. Cajamarca, Peru. Universidad Nacional de Cajamarca Escuela Académico Profesional de Enfermería Facultad Ciencias de la Salud Universidad Nacional de Cajamarca Cajamarca Peru; 2 Facultad de Medicina Humana, Universidad de Piura. Lima, Peru. Universidad Nacional de Piura Facultad de Medicina Humana Universidad de Piura Lima Peru

**Keywords:** Metabolic Syndrome, Adolescent, School Health Services, Schools, Food and Nutrition Education, Primary Prevention, Andean Ecosystem, Andes

## Abstract

**Objective.:**

To estimate the impact of a school-based nutritional and healthy lifestyle intervention to increase the proportions of adolescents free of components of the metabolic syndrome.

**Materials and methods.:**

We conducted a pre-experimental study in a representative sample of adolescents from two schools in a high Andean district of Peru (Cajamarca city); 388 students completed the intervention and the baseline and post-intervention measurement. The intervention had nine thematic modules; each module was developed in an educational session of 45 minutes every two weeks. We used the National Cholesterol Education Program Expert Panel and Adult Treatment Panel III criteria for metabolic syndrome. We used paired proportions comparison (McNemar test) to determine the impact of the intervention.

**Results.:**

At baseline, 20.4% (95% CI: 16.2 to 24.5) students had no metabolic syndrome components, post-intervention this proportion increased to 32,5% (95% CI: 27.7 to 37.3), the difference in proportions was 12.1% (95% CI: 7.5 to 16.8; p<0.001). The prevalence of metabolic syndrome was 4.6% (95% CI: 2.4 to 6.9) at baseline, while post-intervention was 2.3% (95%CI: 0.7 to 3.9). During the analysis of components, the greatest reduction was observed in the proportion of hypertriglyceridemia (difference in proportions: 21.9%, 95%CI: 16.9 to 26.9, p <0.001); we also found a significant reduction in arterial hypertension (difference in proportions: 3.1%, 95% CI: 0.6 to 5.6, p=0.025). In the other components, there were no significant differences.

**Conclusions.:**

The school-based intervention increased in 59.3% the proportion of students free of any component of the metabolic syndrome.

## INTRODUCTION

Worldwide, metabolic syndrome (MS) in the pediatric population is increasing and its prevalence reaches 6.7% in low- and middle-income countries ^1^. This health issue is caused by a combination of factors such as lifestyle changes and eating habits ^2^ ^,^ ^3^, obesogenic environments ^4^, among others. MS in children and adolescents increases the risk of developing MS, carotid intima-media thickening, and type II *diabetes mellitus* in adulthood ^5^; it has also been reported that greater severity of MS in childhood can predict cardiovascular accidents during adulthood ^6^.

Metabolic disorders are frequent in the pediatric population of high Andean areas. In indigenous children in Argentina -residents at an altitude of 3750 m - the prevalence of low HDL cholesterol levels (c-HDL) was 33% ^7^; another study reported that these children from the Argentine Andes had a higher proportion of hypertriglyceridemia compared to children at sea level (28.8% compared to 3.5%) ^8^. These conditions also occur in adolescents from Peruvian high Andean regions. In 2011, the frequency of low c-HDL and hypertriglyceridemia reached 44.8% and 19.4% among schoolchildren in Arequipa, respectively ^9^. In 2014, a study found 3.2% of MS, 46.4% of hypertriglyceridemia, and 37.0% of low c-HDL levels among 11- to 13-year-old schoolchildren in Cajamarca ^10^. This worrisome scenario in the high Andean regions requires the implementation of interventions that contribute to prevent the occurrence of the components of MS.

Nutritional educational interventions have shown to be beneficial in reducing abdominal obesity in children and adolescents ^11^ ^,^ ^12^; in the latter, interventions that combine the nutritional aspect with physical activity reduce the body mass index (BMI) ^13^. We identified 46 systematic reviews (SR) on nutritional interventions in schools, in 39 SR the population was healthy; among these, 30 evaluated the effect on food intake, 21 on intermediate outcomes such as quality of life, anxiety-depression, academic performance, and only six evaluated the effect on outcomes such as MS, overweight-obesity, anthropometric measures, lipid profile, among others ^14^.

Childhood and adolescence are a window of opportunity to establish healthy lifestyles, and educational interventions are valid strategies for this purpose ^2^ ^,^ ^14^ ^-^ ^16^. Randomized clinical trials (RCTs) are the most suitable designs for evaluating the efficacy of preventive or therapeutic interventions. Between 2006 and 2019 only ten RCTs evaluated the efficacy of educational interventions on MS in pediatric population, and only four carried out a nutritional intervention during school hours. These studies had a low risk of bias; however, they did not report their effect on MS and their results have been inconclusive ^11^.

Schools are ideal scenarios for implementing educational interventions, due to their scope and influence on adolescents during their schooling ^17^. However, it is necessary to evaluate the effect in a real scenario of an educational intervention as a preventive strategy for MS. Therefore, the aim of this study was to estimate the effect of an educational intervention based on nutrition and healthy lifestyles to increase the frequency of adolescents free of some component of MS in two schools in an Andean region of Peru.

**KEY MESSAGES**

Motivation for the study: Metabolic disorders are frequent in adolescents in high Andean areas; therefore, educational interventions based on nutrition and healthy lifestyles are required to prevent the occurrence of the components of the metabolic syndrome.

Main findings: The intervention increased the proportion of adolescents free of metabolic syndrome components from 20.4% to 32.5% (difference in proportions: 12.1%, 95%CI: 7.5 to 16.8), this effect was found in both males and females.

Implications: Educational interventions in schools are strategies with a potential protective effect on the occurrence of metabolic syndrome in adolescence..

## MATERIALS AND METHODS

### Study design and setting

The study was carried out in the district of Cajamarca (2750 m altitude) in northeastern Peru. In 2017, the school population aged 12 to 16 years was 118,308; of these, 38,080 were from the urban area, and 74.0% of those aged 12 years and older were considered mestizo ^18^. We conducted a pre-experimental study; dependent variables were measured before and after the intervention in a single study group. We worked in two public schools; in 2019 the “Juan XXIII” school had 1666 high school students, while the “San Ramón” school had 1597, the former only admits females and the latter, males.

### Selection criteria and sample design

The inclusion criteria were: to be high school students of the indicated schools during year 2019; to have given informed consent to participate in the study, and that the father, mother or guardian had given informed consent for the participation of the child. The following were excluded: pregnant women, students with physical limitations that made anthropometry impossible, those who decided to withdraw from the study during the intervention, and those who completed the intervention but did not participate in the subsequent measurement.

The sample size was calculated for a hypothesis of comparison of proportions in paired samples. The outcome was the proportion of students without any component of MS; this baseline proportion was considered to be 39.9%, according to a previous study in Cajamarca ^10^; for the postintervention proportion, we assumed an increase of 20 percentage points (pp) ^17^, with a statistical power of 95% and a significance level of 5%; the size calculated in Epidat 3.1 was 163 participants; this calculation was made for each school to have a comparable and representative group of males and females. For possible losses during follow-up, we considered a non-response rate of 20%. The planned sample size per school was 196 participants.

Each section had between 28 and 30 students, so we selected, on average, seven sections per school. The sections were considered as the primary sampling units. Stratified random sampling with random bootstrap was applied to ensure the selection of one section per year of study (grades 1 to 5). We paired by year of study to control for the effect of age.

### Metabolic syndrome

We used the National Cholesterol Education Program Expert Panel and Adult Treatment Panel III criteria (hereafter NCEP ATP III criteria) adjusted for adolescents ^19^. This criterion defines MS as the presence of three or more components: waist circumference ≥ 90th percentile for age and sex; fasting plasma glucose ≥ 110 mg/dL; systolic or diastolic blood pressure ≥ 90th percentile for age/sex/height; triglycerides ≥ 110 mg/dL; and c-HDL ≤ 40 mg/dL ^1^ ^,^ ^19^. We selected these criteria because they use percentiles of age- and sex-specific reference values for two of its five components ^20^. The reference percentiles for abdominal circumference ^21^ and arterial hypertension (AHT) ^22^ for adolescents were previously established.

### Clinical evaluation and sampling

Anthropometry - weight, height and abdominal perimeter - was performed by a nurse trained in the national technical guidelines for anthropometric assessment of adolescents ^23^ and certified as an anthropometrist by the National Institute of Health (Lima, Peru). Blood pressure was measured by six nurses using Riester exacta® aneroid sphygmomanometers with Velcro cuffs for small adults and Riester Duplex® stethoscopes. Triplicate measurement of systolic and diastolic pressure was performed between 8 and 9 am, prior to blood sampling, with the student seated and at rest for 15 min, with the uncovered right arm resting on a table and flexed at the level of the heart, the inflatable cuff covered two thirds of the length and circumference of the arm. Physical activity was measured with the short version of the International Physical Activity Questionnaire (IPAQ) and its criteria for defining low, moderate and high levels ^24^.

The blood sample was obtained by venipuncture by a medical technologist and with the student fasting. For each participant, 5 mL of whole blood was obtained in a polyethylene terephthalate tube with coagulant activator and gel; samples were then transported to a private laboratory -within the first hour of collection- where they were centrifuged and separated into aliquots for processing on the same day of sampling.

The first measurement was taken in May and June 2019, and the postintervention measurement was in November and December 2019. Anthropometric and blood pressure measurements were performed by trained contract personnel who were not part of the research team; these personnel performed the postintervention measurements blinded to the baseline result. The laboratory processing personnel were independent of the research team and were also blinded to the baseline measurement.

### Laboratory tests

Glucose, total cholesterol and triglyceride levels in serum were determined using the enzymatic method and spectrophotometer readings; the determination of c-HDL was performed with the colorimetric method without precipitation, and LDL cholesterol (c-LDL) was measured by colorimetric method based on a homogeneous assay without precipitation. Wiener reagents and a Wiener model CB 400i automated biochemical analyzer were used in all cases.

### Description of the intervention

The learning sessions were designed by specialists in education, nutrition, health promotion and psychology, based on the proposed model for healthy lifestyles and social learning of the Ministry of Education. The didactic sequence included motivation, collection of previous knowledge, new knowledge and evaluation. We used printed materials, audiovisuals and real objects. The methodology was interactive-participative. The intervention was implemented simultaneously between June and November 2019 and comprised nine thematic modules, each conducted in one educational session (Table 1). The sessions were carried out by six health professionals with experience in child and adolescent health promotion and education. Each professional conducted a demonstration session as a pilot test in a school that was not part of this study. The pilot was used to make adjustments to the dynamics of the sessions, the content provided and the teaching materials. During the intervention period, the students were not exposed to other educational or preventive interventions related to the modules.

**Table 1:** Description of the educational intervention based on healthy nutrition and healthy lifestyles.

N.°	Thematic module of the session ^a^	Description	Goal of the session	Facilitator profile
1	Introduction: Healthy lifestyles	Healthy lifestyles were addressed and the topics were ^a^: Healthy food and nutrition: - Adequate nutritional status. - Healthy food and nutrition. - Food needs during adolescence. - Preparation of healthy meals b) Physical activity c) Rest and sleep d) Recreation e) Emotional control	Promote the importance of healthy lifestyles as a practice to reduce health risks.	Nurse, specialized in public health, with experience in health promotion and education.
2	Adequate nutritional status	Description of the basic concepts of nutritional status, recognition of adequate nutritional status, assessment of nutritional status in adolescents, interpretation of BMI, weight/age indicator and how to stay healthy from very early stages of life.	To promote the importance of an adequate nutritional status in order to stay healthy from the adolescent stage.	Nurse, specialized in education and health promotion. Teaching experience in maternal and child health, and child and adolescent health ^b^
3	Healthy eating and nutrition	Description of the basic concepts of food and nutrition: definition of healthy eating, presentation of the 12 messages of food and nutrition according to the Peruvian food guide, importance of a balanced diet, identification of food groups and types of food: natural, processed and ultra-processed in the diet of adolescents.	Knowledge of the basic concepts of healthy eating and nutrition and their applicability in the daily life.	Nurse, specialized in educational management, planning and administration. Teaching experience in maternal and child health, and child and adolescent health.
4	Nutritional needs in adolescence	Basic concepts of dietary needs, identification of the basic needs of a healthy diet in adolescents, nutritional requirements per day in adolescents, description of foods that meet nutritional needs such as: cereals, tubers; vegetables; fruits; dairy products and derivatives; meats, fish, eggs; sugars; oils and fats. Iron supplementation for adolescent girls between 12 and 17 years of age	Identify the basic food needs of a person in the adolescent stage.	Nurse, specialized in education and health promotion. Teaching experience in maternal and child health, and child and adolescent health ^b^
5	Preparation of healthy meals	Description of the basic concepts on food preparation and consumption; percentage distribution of energy requirement per day. Total 2050 kcal in adolescents aged 12 to 14 years, distributed as 20% for breakfast, 40% for lunch, 10% for mid-afternoon and 30% for dinner. In adolescents aged 15 to 17 years, 2300 kcal with the same distribution percentages. Planning and consumption of healthy menus, food portions by food groups and age.	Identify steps to prepare healthy meals for adolescents, using local ingredients.	Nurse, specialized in public health. Teaching experience in nutrition in nursing.
6	Physical activity	Description of the importance of physical activity to improve the quality of life, importance and benefits of physical activity, frequency, duration, intensity and type of physical activity as well as ways to perform physical activity.	To promote the importance of daily physical activity as a means of preventing chronic diseases in adolescence.	Nurse, specialized in clinical psychology.
7	Rest and sleep	Description of the basic concepts of rest and sleep; importance of rest and sleep; the circadian cycle of sleep and wakefulness; types of sleep, role of sleep in learning and benefits of adequate rest.	Promote the development of individual and collective recreational activities for the well-being of the person.	Licensed in psychology ^c^
8	Recreation	Description of the basic concepts of recreation, importance of recreation, characteristics and benefits of recreational activities and factors that influence recreational activities.	To recognize the importance of rest and sleep for the health of people’s lives, especially in the adolescent stage.	Licensed in psychology ^c^
9	Emotional control	Description of basic concepts of emotional control, importance of emotional control in health, basic or primary emotions, secondary or complex emotions, positive and negative emotions, advantage of emotion control and emotional intelligence.	Identify various feelings, emotions and propose healthy alternatives to control and overcome the negative aspects that affect health.	Licensed in psychology ^c^

a Each session was biweekly and lasted 45 min. Each session covered the following content: informative data, purpose, cross-sectional approach and learning development. The first session was implemented in one school, and in the following week, the same session was conducted in the other school, until the content of the intervention was completed. The sessions were carried out during tutoring hours, in some cases in the courses of education for work or religion. Parents or guardians were not present during the sessions. No printed material was handed out to take home. The instructional material for each thematic module is available at: https://www.unc.edu.pe/escuela-academico-profesional-de-enfermeria/.

b Sessions 2 and 4 were carried out by the same nurse.

c Sessions 7, 8 and 9 were carried out by the same psychologist.

### Statistical analysis

We carried out a descriptive analysis of demographic characteristics, physical activity, nutritional status by anthropometry, lipid profile, serum glucose level, systolic and diastolic blood pressure using frequencies and percentages for categorical variables, and means with standard deviation or median with interquartile range (IQR) according to the type of distribution of the quantitative variable. The entire sample was analyzed descriptively and stratified according to sex. The Kolmorogov-Smirnov test was used to evaluate the distribution of the data.

The frequency of students without MS components (pre- and post-intervention) was expressed as a point (%) and with 95% confidence intervals (95%CI). Measurements for the dependent variables (absence of MS components) were compared with McNemar’s test and difference of proportions (Δp) with 95%CI. We also compared the proportions of MS and its components (McNemar) and medians using the Wilcoxon test for paired data, this analysis only included data from students who completed both measurements. The comparison was made for the entire sample, stratified by sex and by nutritional status (defined from baseline BMI). A value of p ˂0.05 was considered statistically significant. We carried out the analysis in SPSS version 25.

### Ethical aspects

The research protocol, assent and informed consent forms were approved by the Research Ethics Committee of the Universidad Nacional de Cajamarca. All students from the selected sections were invited to participate. The process of student assent and parental consent was carried out simultaneously and at the school facilities. Prior to the second measurement, children and parents underwent a reconsent process. The authorities of both schools provided authorization and facilities for the research. The data were confidential; only the researchers had access to the database. The results of the laboratory tests and anthropometry were given individually to the parents at no cost. Participants did not receive monetary compensation.

## RESULTS

### Baseline characteristics of the sample

A total of 388 students were included, all of whom received the complete sessions of the intervention (Figure 1 of supplementary material). The 18 students excluded after the baseline measurement did not differ from those included according to age, sex and BMI (Table 1 of supplementary material). At baseline measurement, the mean age and standard deviation (SD) was 14.2 (1.7) years; 195 (50.3%) were male; other baseline characteristics are shown in Table 2.

**Figure 1:**
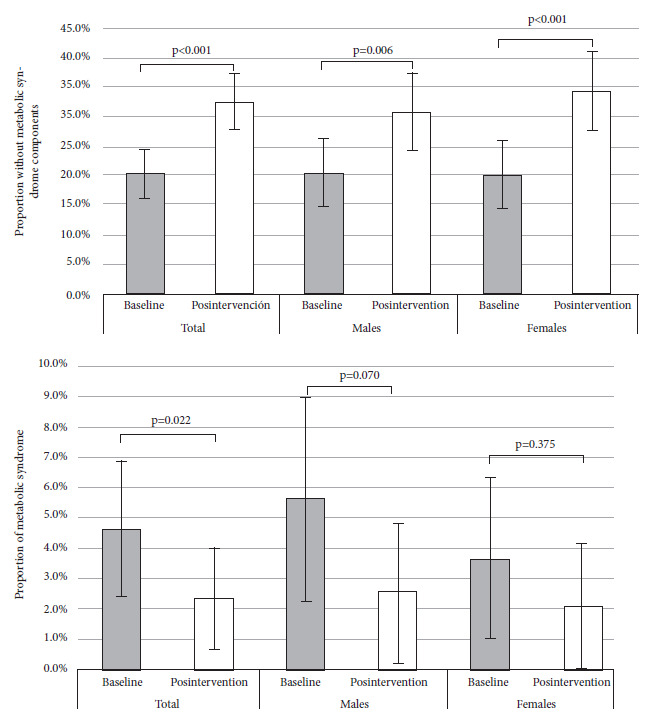
Comparison of the pre- and post-intervention proportion (total and according to sex) of two outcomes in adolescents: absence of metabolic syndrome components (upper) and metabolic syndrome (three or more components) (lower).

**Table 2:** Baseline characteristics of school adolescents who completed both measurements and stratified by sex.

Characteristic	Total (n=388)	Male (n=195)	Female (n=193)	p-value
Abdominal circumference (cm), median (IQR)	72.00 (68.00-78.00)	73.00 (68.00-79.00)	71.00 (67.00-77.50)	0.058^ a^
Triglycerides (mg/dL), median (IQR)	100.00 (76.00-138.75)	96.00 (73.00-133.00)	104.00 (79.50-142.00)	0.078 ^a^
HDL cholesterol (mg/dL), median (IQR)	37.00 (33.00-43.00)	35.00 (30.00-41.00)	38.00 (34.00-45.00)	<0.001^a^
LDL cholesterol (mg/dL), median (IQR)	87.20 (75.10-102.95)	83.00 (70.20-97.20)	92.00 (79.20-108.10)	<0.001^a^
Total cholesterol (mg/dL), median (CI)	147.00 (133.00-168.00)	143.00 (129.00-160.00)	156.00 (137.00-176.00)	<0.001^a^
Fasting plasma glucose (mg/dL), median (IQR)	80.00 (74.00-86.00)	81.00 (74.00-86.00)	80.00 (74.00-85.00)	0.775 ^a^
Nutritional status (BMI), n (%) ^c^				
Normal	245 (63.3)	121 (62.4)	124 (64.2)	0.237 ^b^
Overweight	111 (28.7)	53 (27.3)	58 (30.1)	
Obese	31 (8.0)	20 (10.3)	11 (5.7)	

IQR: interquartile range; BMI: body mass index.

a Mann-Whitney U test

b Chi-square for homogeneity between levels.

c A case of thinness in men was not included in the analysis

### Comparison of the proportion of students without metabolic syndrome components

At baseline measurement, 20.4% (95%CI: 16.2 to 24.5) (n=79) of adolescents had no components of MS, post-intervention the proportion was 32.5% (95%CI: 27.7 to 37.3) (n=126), the increase was 12.1% (95%CI: 7.4 to 16.8, p<0.001) (Table 3). This trend was also found among females (difference in proportions (Δp): 14.0%, 95%CI: 7.6 to 20.4, p<0.001) and males (Δp: 10.3%, 95%CI: 3.4 to 17.1, p=0.006) (Figure 1). Among those with normal nutritional status, the proportion of students without MS components was 25.3% (95%CI: 19.8 to 30.8) (n=62) at baseline measurement and 37.6% (95%CI: 31.4 to 43.7) (n=92) post-intervention; among those overweight, the baseline proportion was 14.4% (95%CI: 7.8 to 21.1) (n=16) and 28.8% (95% CI: 20.3 to 37.4) (n=32) post-intervention; and among those obese, all adolescents had at least one MS component at baseline measurement and 6.5% (95%CI: -2.7 to 15.6) (n=2) had no components post-intervention (Table 4).

**Table 3:** Comparison in paired samples of baseline and post-intervention frequencies of metabolic syndrome components in adolescents.

Components of metabolic syndrome	A	B	C	Frequency in baseline measurement (A+B) (n=388)	Frequency of post-intervention measurement (B+C) (n=388)	Δp (95%CI)	p-value ^a^
No components	22	57	69	79 (20.4)	126 (32.5)	12.1 (7.4 to 16.8) ^b^	<0.001
Abdominal obesity	5	8	0	13 (3.4)	8 (2.1)	1.3 (0.2 to 2.4)	0.063
Arterial hypertension	18	5	6	23 (5.9)	11 (2.8)	3.1 (0.6 to 5.6)	0.025
Low HDL cholesterol	50	205	35	255 (65.7)	240 (61.9)	3.9 (-0.8 to 8.5)	0.129
Hypertriglyceridemia	101	58	16	159 (41.0)	74 (19.1)	21.9 (16.9 to 26.9)	<0.001
Hyperglycemia	1	0	2	1 (0.3)	2 (0.5)	-0.3 (-1.1 to 0.6)	1.000
Metabolic syndrome	11	7	2	18 (4.6)	9 (2.3)	2.3 (0.3 to 3.2)	0.022

A=number of adolescents with presence of a component at baseline and absence of it at postintervention, B=number of adolescents with no change, C=number of adolescents with absence of a component at baseline and with absence of it at postintervention, Δp=difference of proportions (baseline - postintervention), 95%CI=95% confidence interval.

a McNemar’s test (two-tailed test)

b Δp= postintervention - baseline

**Table 4:** Comparison in paired samples of the baseline and post-intervention frequency of metabolic syndrome components according to nutritional status of adolescents ^a^.

Metabolic Syndrome/Component		Normal (n=245)		Overweight (n=111)		Obesity (n=31)	
	Measurement	Postintervention		Postintervention		Postintervention	
	Baseline	No	Yes	No	Yes	No	Yes
Absence of metabolic syndrome components	No	137	46	74	21	29	2
	Yes	16	46	5	11	0	0
	p-value	<0.001		0.002		nc	
	Δp (95%CI) ^b^	12.2 (6.1 to 18.4)		14.4 (5.7 to 23.1)		6.5 (2.7 to 15.6)	
Metabolic syndrome	No	242	0	107	1	18	1
	Yes	2	1	3	0	6	6
	p-value	0.500		0.625		0.125	
	Δp (95%CI)	0.8 (-0.3 to 2.0)		1.8 (-1.8 to 5.4)		16.1 (-0.5 to 32.8)	
Central obesity	No	244	0	111	0	19	0
	Yes	0	1	0	0	5	7
	p-value	1.000		nc		0.063	
	Δp (95%CI)	0		0		16.1 (2.4 to 29.8)	
Arterial hypertension	No	237	3	100	2	21	1
	Yes	5	0	9	0	4	5
	p-value	0.727		0.065		0.375	
	Δp (95%CI)	0.8 (-1.5 to 3.1)		6.3 (0.5 to 12.1)		9.7 (-4.9 to 24.2)	
Hypertriglyceridemia	No	154	9	48	6	10	1
	Yes	57	25	39	18	5	15
	p-value	<0.001		<0.001		0.219	
	Δp (95%CI)	19.6 (13.5 to 25.7)		29.7 (19.1 to 40.4)		12.9 (-2.8 to 28.6)	
Low HDL cholesterol	No	72	24	24	9	2	1
	Yes	33	116	14	64	3	25
	p-value	0.289		0.405		0.625	
	Δp (95%CI)	3.7 (-2.4 to 9.7)		4.5 (-4.1 to 13.1)		6.5 (-6.7 to 19.6)	
Hyperglycemia	No	245	0	109	2	30	0
	Yes	0	0	0	0	1	0
	p-value	nc		nc		nc	
	Δp (95%CI)	0		-1.8 (-4.3 to 0.7)		3.2 (-3.4 to 9.8)	

The p-value corresponds to McNemar’s test (two-tailed test), nc: not calculable, Δp: difference in proportions (baseline - postintervention), 95% CI: 95% confidence interval.

Values in cells correspond to absolute frequencies

a One participant who was thin was not included; nutritional status was constructed from baseline body mass index.

b Δp: postintervention - baseline.

### Comparison of the proportion of metabolic syndrome and its components.

At baseline, the prevalence of MS was 4.6% (95%CI: 2.4 to 6.9), post-intervention it was 2.3% (95%CI: 0.7 to 3.9), the Δp was significant (2.3%, 95%CI: 0.3 to 3.2, p=0.022). In the stratified analysis by sex, we found no significant differences in males (Δp of 3.1%, 95%CI: -0.2 to 4.1) and in females (Δp of 1.6%, 95%CI: -1.1 to 2.6) (Figure 1). In the component analysis, the greatest reduction was in hypertriglyceridemia (Δp of 21.9%, 95%CI: 16.9 to 26.9, p<0.001), a reduction of 3.1% (CI95%: 0.6 to 5.6, p=0.025) was also observed among the proportions of AHT. There were no significant changes in the other components (Table 3).

### Comparison of the components of metabolic syndrome according to sex and nutritional status

According to sex, the proportion of abdominal obesity decreased from 3.1% (6/195) to 2.1% (4/195) (p=0.500) in men and from 3.6% (7/193) to 2.1% (4/193) (p=0.250) in women; AHT was reduced from 8.7% (17/195) to 4.1% (8/195) (p=0.049) in men, and from 3.1% (6/193) to 1.6% (3/193) (p=0.453) in women; hypertriglyceridemia decreased from 36.9% (72/195) to 19.5% (38/195) (p<0.001) in men and from 45.1% (87/193) to 18.7% (36/193) (p<0.001) in women; the proportion of low c-HLD decreased from 70.3% (137/195) to 64.1% (125/195) (p=0.096) in men and from 61.1% (118/193) to 59.6% (115/193) (p=0.755) in women; finally, in men we found no cases of hyperglycemia at baseline measurement, but one post-intervention; among women the proportion remained at 0.5% (1/193) (p=1.00). Table 2 of the supplementary material shows the comparison of the medians of lipid profile, serum glucose, blood pressure and physical activity before and after the intervention, stratified by sex; Table 5 shows the changes in the number of MS components between baseline and post-intervention measurements. The changes in the proportion of MS and its components, according to nutritional status, are shown in Table 4.

**Table 5:**
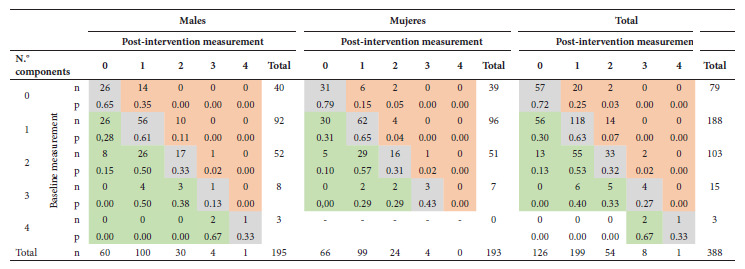
Comparison between the number of metabolic syndrome components at baseline and post-intervention measurement among adolescents, stratified by sex.

n=frequency, p=proportion, McNemar-Bowker test (two-tailed test) in males (p<0.001), females (p<0.001) and total (p<0.001).

Cells shaded in green correspond to participants who decreased the number of components for the post-intervention measurement, cells in gray are those who remained with the same number of components, and cells in red are those who increased the number of components.

Note to facilitate reading of the table: Among 188 adolescents who had one component in the baseline measurement, 56 no longer had it postintervention; among the 92 males with one component, 26 presented such a change, and among the 96 females, 30 also had it. Among 103 students who had two components at baseline, 55 reduced to one component and 13 no longer had two components.

## DISCUSSION

The educational intervention that was implemented in two secondary schools in a high Andean region of Peru achieved two relevant changes; first, an increase of 12 pp in the proportion of adolescents without MS components, and second, a significant reduction in the frequency of MS. After the intervention was completed, 11 of 18 adolescents who initially had MS no longer had having MS. This change could be explained mainly by the effect on hypertriglyceridemia, since 101 of 159 adolescents went from elevated triglyceride values to normal.

Unlike our research, most evaluations of educational interventions with single-group pre- and post-intervention design were conducted in adolescents or overweight children. Two studies were conducted in the educational setting, one in 98 Mexican children aged 6 to 12 years and reported a 44 to 16% reduction in MS, also reduced hypertriglyceridemia (64 to 35%), AHT (19 to 0%), and c-HDL≤ 40 mg/dL (60 to 41%) ^17^. The other study took place in Chile and found that 28 of 50 obese students, who completed an 8-month intervention, achieved a reduction in MS from 36 to 18%; this effect was caused by decreases in glucose, triglyceride, and c-LDL levels ^25^. Other pre-experimental studies-but in non-school settings-show a varied effect in reducing MS. An intervention in 53 children and adolescents in Colombia, from a hospital-based obesity program, reduced MS from 37 to 32% ^26^. A community intervention in 144 obese children and adolescents in the United States found a modest reduction of MS (30.6 to 27.1%) ^27^. On the other hand, a study in 85 obese children from an Italian hospital found a decrease of 17.1 to 4.9% between pre- and postintervention measures ^28^.

Our results are best compared with a study that evaluated the effect of an educational intervention on MS in adolescents from the general population. The study was conducted in Spain in 263 adolescents aged 12 to 16 years and found a significant reduction in MS (32.2 to 19.7%); in hypertriglyceridemia (4.7 to 0.8%), and the in those affected by c-HDL≤40 mg/dL (2.8 to 0.7%) ^29^. Although we observed consistent results regarding the effect of these educational interventions on MS, the size of the effect differs between studies. This variability could be explained by the different types of target population -overweight or obese versus general population-; the implementation setting -schools, hospitals or community-; the duration and dosage of the intervention sessions; the sample size; the criteria for MS -ATP-III, Cook or IDF-, and the baseline prevalence of MS.

Interventions implemented in schools setting differ in duration; in our study, nine group sessions were conducted every 15 days, and each session lasted 45 min. Elizondo *et al*. ^17^ evaluated an intervention for overweight children by means of thirteen individual (not group) sessions every three weeks, during a school year; each session lasted 30 min. This study found that the intervention also reduced BMI and increased physical activity. Campos *et al.* ^29^ designed an intervention for high school students characterized by group sessions every 15 days during the school year; the study did not specify the number or duration of sessions. This intervention also reduced daily calorie intake in males and females. On the other hand, Bustos *et al*. ^25^ evaluated an intervention of 12 nutritional sessions provided during 8 months for obese schoolchildren, with one 40-minute session per week; physical activity sessions of 50 minutes were also carried out twice a week. This study also found an effect on BMI and the percentage of total fat in the trunk and the four extremities.

During the stratified analysis by sex, we found, a significant increase in the proportion of adolescents free of MS components in both males and females; on the other hand, the reduction in the proportion of MS -in both males and females- was not significant. A consistent reduction in hypertriglyceridemia was found in both sexes; this trend was not observed in the study by Campos *et al*. who, among Spanish schoolchildren, found a significant difference in mean triglycerides before and after the intervention only in boys ^29^.

The intervention showed a differentiated effect according to the nutritional status of the adolescent, since no significant effect was found among obese adolescents. This low intensity of response to interventions in obese children and adolescents has been previously reported. This finding could suggest the need for specific interventions to reduce MS in this subgroup ^27^ ^,^ ^29^. Interventions that incorporate physical exercise programs, as well as monitoring of physical activity at home are among the suggested strategies; and these should be carried out in addition to the intervention ^25^ ^,^ ^29^. This study and most research have designed the intervention to be delivered in one school year ^17^ ^,^ ^25^ ^,^ ^27^ ^,^ ^29^, so to improve outcomes in obese individuals, such interventions should be evaluated for more than 12 months.

This intervention produced a significant reduction in the proportion of hypertriglyceridemia, both in the overall sample and in strata defined by sex and nutritional status. A meta-analysis of RCTs to treat overweight and obese children and adolescents found that dietary interventions reduced triglyceride concentration more than 13.3 mg/dL on average, compared to the group that received diet plus exercise program ^12^. A pre-experimental study in Mexican children reduced hypertriglyceridemia (≥110 mg/dL) from 64 to 35% ^17^, whereas in Spanish adolescents the reduction in participants with triglycerides >150 mg/dL was 4.7 to 0.8% ^29^. Both triglycerides and c-LDL are highly correlated with food intake ^30^; therefore, a direct effect of nutritional educational interventions on the dietary habits of adolescents is plausible, generating not only a reduction in triglyceride levels but also in c-LDL. Although overweight and obesity in Latino adolescents is associated with dyslipidemias ^31^ ^,^ ^32^, in this study the prevalence of obesity was only 8%, while that of hypertriglyceridemia was 41.0%; therefore, it is unlikely that the effect on triglycerides is explained by the reduction of excess weight.

The 3.8 pp reduction in the proportion of students with low c-HDL levels was not significant; this finding differs from a study, not restricted to overweight adolescents, in Spanish schoolchildren aged 12 to 16 years in which a significant increase in the mean c-HDL level was found between pre- and post-intervention measurements ^29^. The favorable effect of educational interventions on the increase in c-HDL levels has also been found in pre-experimental studies in overweight adolescents ^17^ ^,^ ^27^ ^,^ ^28^. Similar to our study, Bustos *et al.* reported a decrease in mean c-HDL levels in Chilean children and adolescents ^25^. Our finding could be due to the fact that 35 students with c-HDL values>40 mg/dL at baseline measurement increased to levels ≤40 md/dL postintervention, this phenomenon would correspond to a potential maturation bias, as it has been seen in adolescents aged 10 to 17 years in Brazil, in whom c-HDL levels tend to decrease approaching a mean of 40 mg/dL at older age ^33^.

The limitations of the study are related to the absence of a control group; however, we implemented the intervention for 6 months and the baseline and postintervention measurements were performed at that time, thus reducing the biological maturation effect ^34^ and the loss of participants. During both measurements we did not perform repeated tests for glucose, triglycerides, and c-HDL levels, which could affect the precision of these measurements and thus generate a potential regression bias to the mean, affecting the estimation of the effect of the intervention. To reduce this risk, tests were made on an empty stomach, with adequate pre-analytical treatment of the sample and using standardized and calibrated laboratory procedures, thus reducing instrumentation bias. The intervention was carried out in two schools, one exclusively for females and the other for males; therefore, some differences between the two settings, which were not measured in the study, could confound the effect of the intervention. Intermediate outcomes such as changes in food consumption or level of knowledge about healthy eating were not assessed. Finally, it was not possible to exclude potential cases of primary dyslipidemias, which, depending on their prevalence, could cause selection bias.

In conclusion, the educational intervention on nutrition and healthy lifestyles implemented in two schools in an Andean area of Peru increased the number of adolescents free of MS components, it also caused a significant reduction in the proportion of adolescents with MS, the latter effect being explained mainly by the change in triglyceride levels. We recommend evaluating the effect of the intervention in adolescents from schools in the coast and jungle regions, and it should be adapted to the availability of food in the region, as well as the cultural context.
