# Supplementary material for: Lifestyle school-based intervention to increase the proportion of adolescents free of components of the metabolic syndrome in an andean region of Peru
Source: Rev Peru Med Exp Salud Publica. 2022 Mar 31;39(1):36–46. doi: 10.17843/rpmesp.2022.391.9986 (PMC11397716; doi:10.17843/rpmesp.2022.391.9986)
Supplement: Supplementary material. — Available in the electronic version of the RPMESP. [file rpmesp-39-01-9986-s001.pdf]

MATERIAL SUPLEMENTARIO

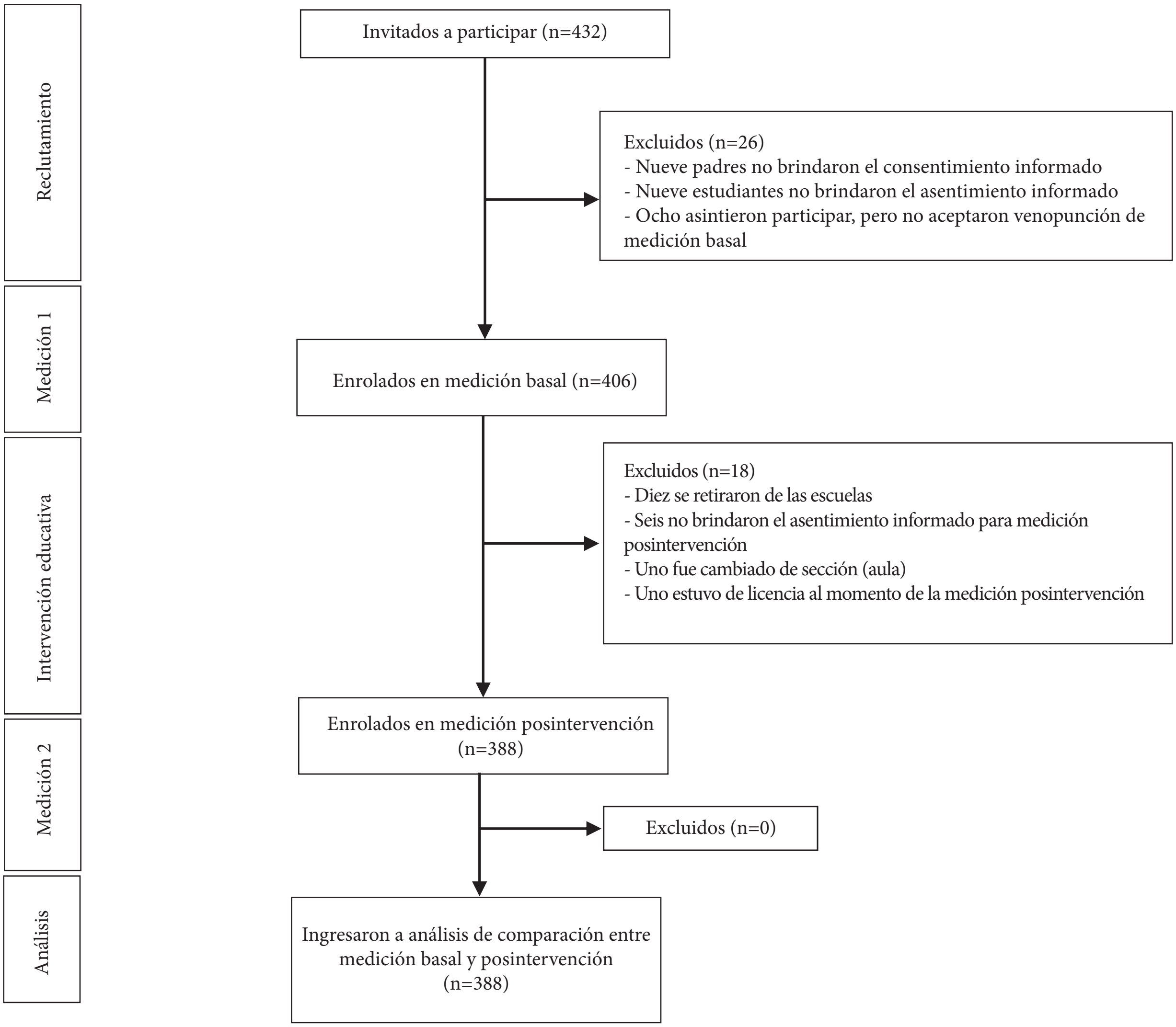

Figura 1. Diagrama de flujo en la selección de los participantes en el diseño del estudio

Rev Peru Med Exp Salud Publica
https://doi.org/10.17843/rpmesp.2022.381.9986

Tabla 1. Comparación de características demográficas y estado nutricional entre estudiantes que cumplieron y no cumplieron con la medición posintervención

Variable	Excluidos (n=18)	Incluidos (n=388)	Valor de p
Edad (años) - Mediana (IC)	15,00 (13,75 – 16,00)	14,00 (13,00 – 16,00)	0,073 [a]
Sexo masculino	10 (55,6)	195 (50,3)	0,843 [b]
Índice de masa corporal – Mediana (IC)	21,84 (20,08 – 23,21)	21,51 (19,71 – 23,90)	0,966 [a]
Valoración nutricional antropométrica (IMC/edad)			
Delgadez	1 (5,6)	1 (0,3)	0,154 [c]
Normal	13 (72,2)	245 (63,1)	
Sobrepeso	3 (16,7)	111 (28,6)	
Obesidad	1 (5,6)	31 (8,0)	
Síndrome metabólico (presencia)	1 (5,6)	18 (4,6)	0,586 [d]

[a] U de Mann-Whitney. [b] Chi cuadrado con corrección de Yates. [c] Chi cuadrado de tendencial lineal. [d] Prueba exacta de Fisher. Todas las pruebas de hipótesis fueron a dos colas.
IC: intervalo intercuartil

Tabla 2. Comparación entre valores de presión arterial, perímetro abdominal, perfil lipídico, glucosa y actividad física de la medición basal y posintervención, estratificado según sexo

Variable	Varones (n=195)			Mujeres (n=193)		
	**Medición basal mediana (IC)**	**Medición final Mediana (IC)**	**Valor de p[a]**	**Medición basal Mediana (IC)**	**Medición final Mediana (IC)**	**Valor de p[a]**
Presión arterial sistólica (mmHg)	100,0 (93,3 a 106,6)	96,6 (90,0 a 100,0)	<0,001	93,3 (90,0 a 101,67)	91,6 (90,0 a 100,0)	<0,001
Presión arterial diastólica (mmHg)	66,6 (60,0 a 70,0)	66,6 (60,0 a 70,0)	0,092	60,0 (60,0 a 70)	60,6 (60,0 a 70,0)	0,517
Perímetro abdominal (cm)	73,0 (68,0 a 79,0)	72,8 (69,1 a 78,1)	0,042	71,0 (67,0 a 77,5)	71.0 (66,1 a 76.0)	<0,001
IMC	21,2 (19,2 a 23,8)	21,3 (19,7 a 23,4)	0,302	21,9 (20,1 a 23,9)	21,9 (20,0 a 23,5)	0,475
Triglicéridos (mg/dL)	96,0 (73,0 a 133,0)	90,0 (69,0 a 106,0)	<0,001	104,0 (79,5 a 142,0)	95,0 (74,5 a 107.0)	<0,001
HDL (mg/dL)	35,0 (30,0 a 41,0)	38,0 (33,0 a 43,0)	<0,001	38,0 (34,0 a 45,0)	39,0 (35,0 a 44,0)	0,427
LDL (mg/dL)	83,0 (70,2 a 97,2)	79,6 (69,0 a 92,2)	<0,001	92,0 (79,2 a 108,1)	89,6 (77,8 a 100,0)	<0,001
Colesterol total (mg/dL)	143,0 (129,0 a 160,0)	138,0 (123,0 a 154,0)	0,001	156.0 (137,0 a 176,0)	149,0 (135,0 a 167.0)	<0,001
Glucosa (mg/dL)	81,0 (74,0 a 86,0)	78,0 (74,0 a 84,0)	0,032	80.0 (74,0 a 85,0)	79,0 (75,0 a 82.0)	1,00
METs–minuto por semana (n=188)	2436,0 (1470,0 a 3519,0)	2946 (2022,0 a 4332,0)	0,001	1194,0 (753,7 a 2346,0)	1950,0 (1251,0 a 4126,0)	<0,001

[a] Comparación de medianas en datos pareados mediante prueba de Wilcoxon (asintótica bilateral).
IC: intervalo intercuartil, IMC: índice de masa corporal.

Rev Peru Med Exp Salud Publica
https://doi.org/10.17843/rpmesp.2022.381.9986
