# Supplementary material for: Lifestyle school-based intervention to increase the proportion of adolescents free of components of the metabolic syndrome in an andean region of Peru
Source: Rev Peru Med Exp Salud Publica. 2022 Mar 31;39(1):36–46. doi: 10.17843/rpmesp.2022.391.9986 (PMC11397716; doi:10.17843/rpmesp.2022.391.9986)
Supplement: Translation

# Translation: Intervención educativa basada en estilos de vida para incrementar la proporción de adolescentes libres de componentes del síndrome metabólico en una región altoandina del Perú

**Authors:** Segunda Aydeé García Flores, Juana Aurelia Ninatanta-Ortiz, Martha Vicenta Abanto Villar, Katia Maribel Pérez Cieza, Rosa Ricardina Chávez Farro, Sara Elizabeth Palacios Sánchez, Franco Ronald Romaní Romaní

**Affiliations:** 1 Escuela Académico Profesional de Enfermería, Facultad Ciencias de la Salud, Universidad Nacional de Cajamarca. Cajamarca, Perú.; 2 Facultad de Medicina Humana, Universidad de Piura. Lima, Perú.

**Keywords:** Síndrome Metabólico, Adolescente, Promoción de la Salud Escolar, Escuela, Educación Alimentaria y Nutricional, Prevención Primaria, Andes

## Abstract

**Objetivo.:**

Estimar el efecto de una intervención educativa basada en nutrición y estilos de vida saludables para incrementar la proporción de adolescentes libres de componentes del síndrome metabólico.

**Materiales y métodos.:**

Se realizó un estudio preexperimental en una muestra representativa de adolescentes de dos escuelas de un distrito altoandino del Perú (ciudad de Cajamarca); 388 estudiantes completaron la intervención, así como la medición basal y la posintervención. La intervención tuvo nueve ejes temáticos, cada uno fue desarrollado en una sesión educativa de 45 min cada dos semanas. Se usó los criterios del National Cholesterol Education Program Expert Panel and Adult Treatment Panel III para el síndrome metabólico. Se realizó la comparación de proporciones pareadas (prueba de McNemar) para determinar el impacto de la intervención.

**Resultados.:**

En la medición basal, el 20,4% (IC95%: 16,2 a 24,5) no tenía componentes de SM, posintervención dicha proporción se incrementó a 32,5% (IC95%: 27,7 a 37,3), la diferencia de proporciones fue 12,1% (IC95%: 7,5 a 16,8; p<0,001). La prevalencia de síndrome metabólico fue 4,6% (IC95%: 2,4 a 6,9) basalmente; posintervención fue 2,3% (IC95%: 0,7 a 3,9). La diferencia de proporciones fue 2,3%, (IC95%: 0,3 a 3,2; p=0,022). Entre los componentes, la mayor reducción fue en la proporción de hipertrigliceridemia (diferencia de proporciones: 21,9%, IC95%: 16,9 a 26,9; p<0,001), también se observó una reducción significativa de la hipertensión arterial (diferencia de proporciones: 3,1%, IC95%: 0,6 a 5,6; p=0,025). En los demás componentes no hubo diferencias significativas.

**Conclusiones.:**

La intervención educativa incrementó en un 59,3% la proporción de estudiantes libres de algún componente del síndrome metabólico.

## INTRODUCCIÓN

A nivel mundial el síndrome metabólico (SM) en población pediátrica está en incremento y su prevalencia llega al 6,7% en países de ingresos bajos y medios ^1^. Este problema sanitario es generado por una combinación de causas como los cambios en los estilos de vida y hábitos alimentarios ^2^ ^,^ ^3^, los entornos obesogénicos ^4^, entre otros. El SM en niños y adolescentes incrementa el riesgo para desarrollar SM, engrosamiento de la íntima media carotidea y diabetes mellitus tipo II en la adultez ^5^, también se ha determinado que una mayor severidad del SM en la infancia predice un accidente cardiovascular en la adultez ^6^. 

Los trastornos metabólicos son frecuentes en población pediátrica de zonas altoandinas. En niños indígenas de Argentina -residentes a 3750 m de altitud- la prevalencia de niveles bajos de colesterol HDL (c-HDL) fue del 33% ^7^, otro análisis reportó que estos niños de los Andes argentinos tenían mayor proporción de hipertrigliceridemia respecto a niños del nivel del mar (28,8% comparado con 3,5%) ^8^. Estas alteraciones también se dan en adolescentes de regiones altoandinas peruanas. En 2011 entre escolares de Arequipa, la frecuencia de c-HDL bajo e hipertrigliceridemia alcanzó el 44,8 y 19,4%, respectivamente ^9^. En 2014, entre escolares de 11 a 13 años de Cajamarca se encontró 3,2% de SM, 46,4% de hipertrigliceridemia y 37,0% de niveles bajos de c-HDL ^10^. Este preocupante escenario en las regiones altoandinas requiere de la implementación de intervenciones que contribuyan a prevenir la ocurrencia de los componentes del SM.

Las intervenciones educativas nutricionales han mostrado beneficio para reducir la obesidad abdominal en niños y adolescentes ^11^ ^,^ ^12^; en estos últimos las intervenciones que combinan el aspecto nutricional con la actividad física reducen el índice de masa corporal (IMC) ^13^. Se han identificado 46 revisiones sistemáticas (RS) sobre intervenciones nutricionales en el ámbito escolar, en 39 RS la población fue sana; entre estas, 30 evaluaron el efecto sobre la ingesta de alimentos, 21 en desenlaces intermedios como calidad de vida, ansiedad-depresión, desempeño académico, y solo seis evaluaron el efecto en desenlaces como SM, sobrepeso-obesidad, medidas antropométricas, perfil lipídico, entre otros ^14^. 

La infancia y la adolescencia constituyen una ventana de oportunidad para establecer estilos de vida saludables, y las intervenciones educativas son estrategias válidas para dicho fin ^2^ ^,^ ^14^ ^-^ ^16^. Los ensayos clínicos aleatorizados (ECA) son los diseños más indicados para evaluar la eficacia de intervenciones preventivas o terapéuticas. Entre el 2006 y 2019 solo diez ECA evaluaron la eficacia de las intervenciones educativas sobre el SM en población pediátrica, y solo cuatro realizaron la intervención nutricional en el horario escolar. Estos estudios presentaron un bajo riesgo de sesgo; sin embargo, no reportaron su efecto sobre el SM y sus resultados no han sido concluyentes ^11^.

Las escuelas son escenarios idóneos para implementar intervenciones educativas, debido a su alcance e influencia en los adolescentes durante la formación escolar ^17^. No obstante, es necesario evaluar en un escenario real el efecto de una intervención educativa como estrategia preventiva del SM. Por lo descrito, el objetivo del estudio fue estimar el efecto de una intervención educativa basada en nutrición y estilos de vida saludables para incrementar la frecuencia de adolescentes libres de algún componente de SM en dos escuelas de una región andina del Perú.

**MENSAJES CLAVE**

Motivación para realizar el estudio: las alteraciones metabólicas son frecuentes en adolescentes de zonas altoandinas; por ello, se requieren intervenciones educativas basadas en nutrición y estilos de vida saludable para prevenir la ocurrencia de los componentes del síndrome metabólico.

Principales hallazgos: la intervención incrementó la proporción de adolescentes libres de componentes del síndrome metabólico del 20,4 al 32,5% (diferencia de proporciones: 12,1%, IC95%: 7,5 a 16,8), dicho efecto se encontró en varones y mujeres.

Implicancias: las intervenciones educativas en el ámbito escolar son estrategias de potencial efecto protector para la ocurrencia del síndrome metabólico en la adolescencia.

## MATERIALES Y MÉTODOS

### Diseño y ámbito de estudio

El estudio fue realizado en el distrito de Cajamarca (2750 m de altitud) al noreste del Perú. En 2017, la población escolar de 12 a 16 años era de 118 308; de ellos, 38 080 eran de la zona urbana, y el 74,0% de los mayores de 12 años se consideraba mestizo ^18^. Realizamos un estudio preexperimental; se midieron las variables dependientes antes y después de la intervención en un solo grupo de estudio. Trabajamos en dos escuelas públicas; en el 2019 el Colegio «Juan XXIII» tenía 1666 estudiantes de secundaria, en tanto que el Colegio «San Ramón» tenía 1597, el primero solo admite mujeres y el segundo, varones.

### Criterios de selección y diseño muestral

Los criterios de inclusión fueron: ser estudiantes de secundaria de los colegios señalados, en el año escolar 2019; haber brindado el asentimiento informado para participar en el estudio, y que el padre, madre o apoderado haya brindado el consentimiento informado para la participación del menor. Fueron excluidas gestantes, estudiantes con alguna limitación física que imposibilite la antropometría, aquellos que decidieron retirarse del estudio durante la intervención y aquellos que culminaron la intervención, pero no participaron de la medición posterior.

El tamaño muestral fue calculado para una hipótesis de comparación de proporciones en muestras pareadas. El desenlace fue la proporción de estudiantes sin ningún componente de SM, se consideró que esta proporción basal fuera 39,9%, según estudio previo en Cajamarca ^10^; para la proporción posintervención se asumió un incremento de 20 puntos porcentuales (pp) ^17^, con una potencia estadística del 95% y un nivel de significancia del 5%; el tamaño calculado en Epidat 3.1 fue 163 participantes, dicho cálculo se hizo en cada escuela para tener un grupo comparable y representativo de varones y mujeres. Por posibles pérdidas durante el seguimiento consideramos una tasa de no respuesta del 20%. El tamaño muestral planeado por escuela fue de196 participantes.

Cada sección tuvo entre 28 y 30 alumnos, por ello, seleccionamos, en promedio, siete secciones por escuela. Las secciones fueron consideradas como unidades primarias de muestreo. Se aplicó un muestreo aleatorio estratificado con arranque aleatorio, así se aseguró la selección de una sección por año de estudio (1.^o^ a 5.^o^ grado). Realizamos un pareamiento según año de estudio para controlar el efecto de la edad.

### Síndrome metabólico

Usamos los criterios del National Cholesterol Education Program Expert Panel and Adult Treatment Panel III (en adelante criterios NCEP ATP III) ajustados para adolescentes ^19^. Este criterio define como SM la presencia de tres o más componentes: circunferencia de cintura ≥ percentil 90 para edad y sexo; glucosa plasmática en ayunas ≥ 110 mg/dL; presión arterial sistólica o diastólica ≥ percentil 90 para edad/sexo/talla; triglicéridos ≥ 110 mg/dL, y c-HDL ≤ 40 mg/dL ^1^ ^,^ ^19^. Seleccionamos estos criterios porque usa percentiles de valores de referencia específicos para edad y sexo en dos de sus cinco componentes ^20^. Los percentiles de referencia para la circunferencia abdominal ^21^ e hipertensión arterial (HTA) ^22^ para adolescentes fueron previamente establecidos.

### Evaluación clínica y toma de muestra

La antropometría -peso, talla y perímetro abdominal- fue realizada por una enfermera entrenada en las guías técnicas nacionales de valoración antropométrica del adolescente ^23^ y certificada como antropometrista por el Instituto Nacional de Salud (Lima, Perú).

La presión arterial fue medida por seis enfermeras que usaron tensiómetros aneroides Riester exacta® con brazalete de velcro para adultos pequeños y estetoscopios Riester Duplex®. La medición por triplicado de la presión sistólica y diastólica fue realizada entre las 8 y 9 de la mañana, previa a la toma de muestra de sangre, con el estudiante sentado y en reposo por 15 min, con el brazo derecho descubierto apoyado en una mesa y flexionado a la altura del corazón, el manguito inflable cubrió dos terceras partes del largo y circunferencia del brazo. Respecto a la actividad física se midió con el cuestionario internacional de actividad física (IPAQ) versión corta y sus criterios para definir los niveles bajo, moderado y alto ^24^.

La muestra sanguínea fue obtenida por venopunción por un tecnólogo médico y con el estudiante en ayunas. Por cada participante se obtuvo 5 mL de sangre total en tubo de polietileno tereftalato con activador coagulante y gel; luego, las muestras fueron transportadas a un laboratorio privado -dentro de la primera hora de la toma- en donde fueron centrifugadas y separadas en alícuotas para su procesamiento el mismo día de la toma de muestra.

La primera medición se realizó en mayo y junio de 2019, en tanto que la medición posintervención fue en noviembre y diciembre del mismo año. Las mediciones antropométricas y de presión arterial fueron realizadas por personal entrenado contratado que no fue parte del equipo de investigación, este personal realizó las mediciones posintervención cegados para el resultado basal. El personal para el procesamiento de laboratorio fue independiente del equipo de investigadores y también estuvo cegado para la medición basal.

### Pruebas de laboratorio

La determinación de los niveles de glucosa, colesterol total y triglicéridos en suero se realizó con el método enzimático y con lecturas en espectrofotómetro; la determinación del c-HDL fue realizada con el método colorimétrico sin precipitación, y el colesterol LDL (c-LDL) se midió mediante método colorimétrico basado en un ensayo homogéneo sin precipitación. En todos los casos se usó reactivos de la marca Wiener y un analizador automatizado bioquímico Wiener modelo CB 400i.

### Descripción de la intervención

Las sesiones de aprendizaje fueron diseñadas por especialistas en educación, nutrición, promoción para la salud y psicología, basados en el modelo propuesto para los estilos de vida saludable y aprendizaje social del Ministerio de Educación. La secuencia didáctica comprendió la motivación, el recojo de saberes previos, nuevo conocimiento y la evaluación. Usamos materiales impresos, audiovisuales y objetos reales. La metodología fue interactiva-participativa. La intervención fue implementada simultáneamente entre junio y noviembre de 2019. La intervención comprendió nueve ejes temáticos, cada uno realizado en una sesión educativa (Tabla 1). Las sesiones fueron conducidas por seis profesionales de la salud con experiencia en promoción y educación para la salud del niño y adolescente. Cada profesional realizó una sesión demostrativa como prueba piloto en una escuela que no formó parte del presente estudio. El piloto sirvió para realizar ajustes en la dinámica de las sesiones, en los contenidos brindados y materiales didácticos. En el periodo de ejecución de la intervención, los estudiantes no estuvieron expuestos a otras intervenciones educativas o preventivas relacionadas con los ejes temáticos brindados.

**Tabla 1:** Descripción de la intervención educativa basada en nutrición saludable y estilos de vida saludables.

N.°	Eje temático de sesión ^a^	Descripción	Objetivo de la sesión	Perfil del facilitador
1	Introducción: Estilos de vida saludable	Se abordó los estilos de vida saludable, teniendo como ejes temáticos a: Alimentación y nutrición saludable: • Estado nutricional adecuado. • Alimentación y nutrición saludable. • Necesidades de alimentación en la adolescencia • Preparación de menús saludables b) Actividad física c) Descanso y sueño d) Recreación e) Control emocional	Promover la importancia de los estilos de vida saludables como práctica para reducir riegos en la salud.	Enfermera, con especialidad en salud pública, y experiencia en promoción y educación para la salud
2	Estado nutricional adecuado	Descripción de los conceptos básicos de estado nutricional, reconocimiento de un estado nutricional adecuado, evaluación del estado nutricional en los adolescentes, interpretación del IMC, indicador peso/edad y como mantenerse saludable desde etapas muy tempranas de la vida.	Fomentar la importancia de un estado nutricional adecuado para mantenerse saludable desde la etapa adolescente	Enfermera, con especialidad en educación y promoción de la salud. Experiencia docente en el área materno infantil, y salud del niño y adolescente ^b^
3	Alimentación y nutrición saludable	Descripción de los conceptos básicos de alimentación y nutrición. ¿Qué es alimentarse saludablemente?, presentación de los 12 mensajes de la alimentación y nutrición según la guía alimentaria peruana, importancia de la alimentación balanceada, identificación de grupos de alimentos y tipos de alimentos: naturales, procesados y ultra procesados en la alimentación de los adolescentes.	Conocer los conceptos básicos sobre alimentación y nutrición saludable y su aplicabilidad en su vida diaria.	Enfermera, con especialidad en gestión, planificación y administración de la educación. Experiencia docente en el área materno infantil, y salud del niño y adolescente
4	Necesidades de alimentación en la adolescencia	Conceptos básicos de las necesidades alimentación, identificación de las necesidades básicas de una alimentación saludable en la adolescencia, requerimientos nutricionales por día en los adolescentes, descripción de alimentos que cubren las necesidades nutricionales como: cereales, tubérculos y menestras; verduras; frutas; lácteos y derivados; carnes, pescado, huevo; azúcares; aceites y grasas Suplementación con hierro a mujeres adolescentes de 12 a 17 años	Identificar las necesidades básicas de alimentación que necesita una persona en la etapa adolescente.	Enfermera, con especialidad en educación y promoción de la salud. Experiencia docente en el área materno infantil, y salud del niño y adolescente ^b^
5	Preparación de menús saludables	Descripción de los conceptos básicos sobre la preparación y consumo de alimentos; distribución porcentual del requerimiento de energía por día. Total de 2050 kcal en adolescentes de 12 a 14 años, distribuidos en 20% en desayuno, 40% en almuerzo, 10% en media tarde y 30% en cena. En adolescentes de 15 a 17 años, 2300 kcal con los mismos porcentajes de distribución. Planificación y consumo de menús saludables, porciones de alimentos por grupos alimentario y edad.	Identificar los pasos para preparar menús saludables para adolescentes, utilizando productos de la zona.	Enfermera con especialidad en salud pública. Experiencia docente en nutrición en enfermería.
6	Actividad física	Descripción la importancia de la actividad física para mejorar la calidad de vida, importancia y beneficios de la actividad física, frecuencia, duración, intensidad y tipo de actividad física y formas de realizar actividad física.	Fomentar la importancia de la actividad física diaria como medio de prevención de enfermedades crónicas en la adolescencia.	Enfermera con especialidad en psicología clínica.
7	Descanso y sueño	Descripción de los conceptos básicos de descanso y sueño; importancia del descanso y sueño; el ciclo circadiano del sueño y la vigilia; tipos de sueño, papel del sueño en el aprendizaje y beneficios del descanso adecuado.	Promover el desarrollo de actividades recreativas individual y colectiva para un bienestar íntegro de la persona.	Licenciada en psicología^c^
8	Recreación	Descripción de los conceptos básicos de la recreación, importancia de la recreación, características y beneficios de las actividades recreacionales y factores que influyen en las actividades recreativas.	Reconocer la importancia del descanso y sueño, para la salud en la vida de las personas, en especial en la etapa adolescente.	Licenciada en psicología^c^
9	Control emocional	Descripción de conceptos básicos de control emocional, importancia del control emocional en la salud, emociones básicas o primarias, emociones secundarias o complejas, emociones positivas y negativas, ventaja del control de emociones e inteligencia emocional.	Identificar diversos sentimientos, emociones y proponer alternativas saludables para controlar y superar los aspectos negativos que afectan la salud.	Licenciada en psicología^c^

a Cada sesión fue bisemanal y duró 45 min. Cada sesión desarrolló el siguiente contenido: datos informativos, propósito, enfoque transversal y desarrollo de aprendizajes. La primera fue implementada en una escuela, y en la semana siguiente la misma sesión fue realizada en la otra escuela, hasta culminar el contenido de la intervención. Las sesiones fueron realizadas en el horario de tutoría, en algunos casos en los cursos de educación para el trabajo o religión. Los padres o tutores no estuvieron presentes durante las sesiones. No se entregó material impreso para llevar al hogar. El material instructivo para cada eje temático se encuentra disponible en: https://www.unc.edu.pe/escuela-academico-profesional-de-enfermeria/

b Las sesiones de los ejes temáticos 2 y 4 fueron realizadas por la misma enfermera.

c Las sesiones 7, 8 y 9 fueron realizadas por la misma psicóloga.

### Análisis estadístico

Realizamos un análisis descriptivo de las características demográficas, actividad física, estado nutricional por antropometría, perfil lipídico, nivel de glucosa sérica, presión arterial sistólica y diastólica utilizando frecuencias y porcentajes para las variables categóricas, y medias con desviación estándar o mediana con intervalo intercuartil (IC) de acuerdo con el tipo de distribución de la variable cuantitativa. El análisis descriptivo se realizó para toda la muestra y estratificado según sexo. Para evaluar la distribución de los datos usamos la prueba Kolmorogov-Smirnov.

La frecuencia de estudiantes sin componentes de SM (pre y posintervención) fue expresada de manera puntual (%) y con intervalos de confianza al 95% (IC95%). Las comparaciones entre las mediciones para la variable dependiente (ausencia de componentes de SM) fueron realizadas con la prueba de McNemar y la diferencia de proporciones (Δp) con IC95%. También comparamos las proporciones de SM y sus componentes (McNemar) y las medianas mediante la prueba de Wilcoxon para datos pareados, este análisis solo incluyó a los datos de estudiantes que completaron ambas mediciones. La comparación se hizo para toda la muestra, estratificada por sexo y por estado nutricional (definida a partir de IMC basal). Un valor de p ˂0,05 fue considerado como estadísticamente significativo. Realizamos el análisis en el programa SPSS versión 25.

### Aspectos éticos

El protocolo de investigación, y los formatos de asentimiento y consentimiento informado fueron aprobados por el Comité de Ética en Investigación de la Universidad Nacional de Cajamarca. Invitamos a participar a todos los estudiantes de las secciones seleccionadas. El proceso de asentimiento de los estudiantes y de consentimiento de los padres fueron realizado de manera simultánea y en las instalaciones escolares. Antes de la segunda medición se realizó un proceso de reconsentimiento a niños y padres. Las autoridades de ambas escuelas brindaron la autorización y facilidades para la investigación. Los datos fueron confidenciales, solo los investigadores accedieron a la base de datos. Los resultados de los exámenes de laboratorio y de la antropometría fueron entregados individualmente a los padres sin costo. Los participantes no recibieron compensación monetaria.

## RESULTADOS

### Características basales de la muestra

Fueron incluidos 388 estudiantes, todos recibieron la intervención con sus sesiones completas (Figura 1 de material suplementario). Los 18 estudiantes excluidos después de la medición basal no tuvieron diferencias con los incluidos según edad, sexo e IMC (Tabla 1 de material suplementario). En la medición basal, la edad promedio y desviación estándar (DE) fue 14,2 (1,7) años; 195 (50,3%) fueron varones; otras características basales se muestran en la Tabla 2.

**Figura 1:** Comparación de la proporción pre y posintervención (total y según sexo) de dos desenlaces en adolescentes: ausencia de componentes de síndrome metabólico (superior) y síndrome metabólico (tres o más componentes) (inferior).

**Tabla 2:** Características basales de los adolescentes escolares que completaron ambas mediciones y estratificado según sexo.

Característica	Total (n=388)	Varones (n=195)	Mujeres (n=193)	Valor de p
Perímetro abdominal (cm), mediana (IC)	72,00 (68,00-78,00)	73,00 (68,00-79,00)	71,00 (67,00-77,50)	0,058^ a^
Triglicéridos (mg/dL), mediana (IC)	100,00 (76,00-138,75)	96,00 (73,00-133,00)	104,00 (79,50-142,00)	0,078 ^a^
Colesterol HDL (mg/dL), mediana (IC)	37,00 (33,00-43,00)	35,00 (30,00-41,00)	38,00 (34,00-45,00)	<0,001^a^
Colesterol LDL (mg/dL), mediana (IC)	87,20 (75,10-102,95)	83,00 (70,20-97,20)	92,00 (79,20-108,10)	<0,001^a^
Colesterol total (mg/dL), mediana (IC)	147,00 (133,00-168,00)	143,00 (129,00-160,00)	156,00 (137,00-176,00)	<0,001^a^
Glucosa plasmática en ayunas (mg/dL), mediana (IC)	80,00 (74,00-86,00)	81,00 (74,00-86,00)	80,00 (74,00-85,00)	0,775 ^a^
Estado nutricional (IMC), n (%)^ c^				
Normal	245 (63,3)	121 (62,4)	124 (64,2)	0,237 ^b^
Sobrepeso	111 (28,7)	53 (27,3)	58 (30,1)	
Obesidad	31 (8,0)	20 (10,3)	11 (5,7)	

IC: intervalo intercuartil, IMC: índice de masa corporal

a U de Mann-Whitney.

b Chi cuadrado de homogeneidad entre niveles.

c No se incluyó en el análisis un caso de delgadez en varones.

### Comparación de la proporción de estudiantes sin componentes de síndrome metabólico

En la medición basal, el 20,4% (IC95%: 16,2 a 24,5) (n=79) de adolescentes no tenía componentes de SM, posintervención dicha proporción fue de 32,5% (IC95%: 27,7 a 37,3) (n=126), el incremento fue de 12,1% (IC95%: 7,4 a 16,8; p<0,001) (Tabla 3). Dicha tendencia se mantuvo entre mujeres (diferencia de proporciones (Δp): 14,0%, IC95%: 7,6 a 20,4, p<0,001) y varones (Δp: 10,3%, IC95%: 3,4 a 17,1, p=0,006) (Figura 1). Entre aquellos con estado nutricional normal, la proporción de estudiantes sin componentes de SM fue 25,3% (IC95%: 19,8 a 30,8) (n=62) en la medición basal y 37,6% (IC95%: 31,4 a 43,7) (n=92) posintervención, entre aquellos con sobrepeso la proporción basal fue 14,4% (IC95%: 7,8 a 21,1) (n=16) y 28,8% (IC95%: 20,3 a 37,4) (n=32) posintervención, y entre los obesos todos los adolescentes tuvieron al menos un componente de SM en la medición basal y 6,5% (IC95%: -2,7 a 15,6) (n=2) no tuvieron componentes posintervención (Tabla 4).

**Tabla 3:** Comparación en muestras pareadas de las frecuencias basal y posintervención de los componentes del síndrome metabólico en adolescentes

Componentes de síndrome metabólico	A	B	C	Frecuencia en medición basal (A+B) (n=388)	Frecuencia en medición posintervención (B+C) (n=388)	Δp (IC95%)	Valor de p^a^
Sin componentes	22	57	69	79 (20,4)	126 (32,5)	12,1 (7,4 a 16,8) ^b^	<0,001
Obesidad abdominal	5	8	0	13 (3,4)	8 (2,1)	1,3 (0,2 a 2,4)	0,063
Hipertensión arterial	18	5	6	23 (5,9)	11 (2,8)	3,1 (0,6 a 5,6)	0,025
Colesterol HDL bajo	50	205	35	255 (65,7)	240 (61,9)	3,9 (-0,8 a 8,5)	0,129
Hipertrigliceridemia	101	58	16	159 (41,0)	74 (19,1)	21,9 (16,9 a 26,9)	<0,001
Hiperglicemia	1	0	2	1 (0,3)	2 (0,5)	-0,3 (-1,1 a 0,6)	1,000
Síndrome metabólico	11	7	2	18 (4,6)	9 (2,3)	2,3 (0,3 a 3,2)	0,022

A= número de adolescentes con componente presente en la medición basal y ausente en la posintervención, B= número de adolescentes sin cambios, C=número de adolescentes con componente ausente en la medición basal y presente en la medición posintervención, Δp= diferencia de proporciones (basal - posintervención), IC95%=intervalo de confianza al 95%.

a Prueba de McNemar (prueba a dos colas)

b Δp=posintervención - basal

**Tabla 4:** Comparación en muestras pareadas de la frecuencia basal y posintervención de los componentes del síndrome metabólico según estado nutricional de adolescentes ^a^

Síndrome Metabólico/Componente		Normal (n=245)		Sobrepeso (n=111)		Obesidad (n=31)	
	Medición	Posintervención		Posintervención		Posintervención	
	Basal	No	Si	No	Si	No	Si
Ausencia de componentes de síndrome metabólico	No	137	46	74	21	29	2
	Sí	16	46	5	11	0	0
	Valor de p	<0,001		0,002		nc	
	Δp (IC95%)^b^	12,2 (6,1 a 18,4)		14,4 (5,7 a 23,1)		6,5 (2,7 a 15,6)	
Síndrome metabólico	No	242	0	107	1	18	1
	Sí	2	1	3	0	6	6
	Valor de p	0,500		0,625		0,125	
	Δp (IC95%)	0,8 (-0,3 a 2,0)		1,8 (-1,8 a 5,4)		16,1 (-0,5 a 32,8)	
Obesidad central	No	244	0	111	0	19	0
	Sí	0	1	0	0	5	7
	Valor de p	1,000		nc		0,063	
	Δp (IC95%)	0		0		16,1 (2,4 a 29,8)	
Hipertensión arterial	No	237	3	100	2	21	1
	Sí	5	0	9	0	4	5
	Valor de p	0,727		0,065		0,375	
	Δp (IC95%)	0,8 (-1,5 a 3,1)		6,3 (0,5 a 12,1)		9,7 (-4,9 a 24,2)	
Hipertrigliceridemia	No	154	9	48	6	10	1
	Sí	57	25	39	18	5	15
	Valor de p	<0,001		<0,001		0,219	
	Δp (IC95%)	19,6 (13,5 a 25,7)		29,7 (19,1 a 40,4)		12,9 (-2,8 a 28,6)	
Colesterol HDL bajo	No	72	24	24	9	2	1
	Sí	33	116	14	64	3	25
	Valor de p	0,289		0,405		0,625	
	Δp (IC95%)	3,7 (-2,4 a 9,7)		4,5 (-4,1 a 13,1)		6,5 (-6,7 a 19,6)	
Hiperglicemia	No	245	0	109	2	30	0
	Sí	0	0	0	0	1	0
	Valor de p	nc		nc		nc	
	Δp (IC95%)	0		-1,8 (-4,3 a 0,7)		3,2 (-3,4 a 9,8)	

El valor de p corresponde a la prueba de McNemar (prueba a dos colas), nc: no calculable, Δp: diferencia de proporciones (basal - posintervención), IC95%: intervalo de confianza al 95%.

Los valores de las celdas corresponden a frecuencias absolutas.

a No se incluyó un participante con delgadez, el estado nutricional fue construido a partir del índice de masa corporal basal.

b Δp: posintervención - basal

### Comparación de la proporción de síndrome metabólico y sus componentes

A nivel basal, la prevalencia de SM fue 4,6% (IC95%: 2,4 a 6,9), posintervención fue 2,3% (IC95%: 0,7 a 3,9), la Δp fue significativa (2,3%, IC95%: 0,3 a 3,2, p=0,022). En el análisis estratificado por sexo no encontramos diferencias significativas en varones (Δp de 3,1%, IC95%: -0,2 a 4,1) y en mujeres (Δp de 1,6%, IC95%: -1,1 a 2,6) (Figura 1). En el análisis de componentes, la mayor reducción se dio en la hipertrigliceridemia (Δp del 21,9%, IC95%: 16,9 a 26,9, p<0,001), también se observó una reducción del 3,1% (IC95%: 0,6 a 5,6, p=0,025) entre las proporciones de HTA. En los demás componentes no hubo cambios significativos (Tabla 3).

### Comparación de los componentes de síndrome metabólico según sexo y estado nutricional

Según sexo, la proporción de obesidad abdominal disminuyó de 3,1% (6/195) a 2,1% (4/195) (p=0,500) en varones y de 3,6% (7/193) a 2,1% (4/193) (p=0,250) en mujeres; la HTA se redujo de 8,7% (17/195) a 4,1% (8/195) (p=0,049) en varones, y de 3,1% (6/193) a 1,6% (3/193) (p=0,453) en mujeres; la hipertrigliceridemia disminuyó de 36,9% (72/195) a 19,5% (38/195) (p<0,001) en varones y de 45,1% (87/193) a 18,7% (36/193) (p<0,001) en mujeres; la proporción de c-HLD bajo disminuyó de 70,3% (137/195) a 64,1% (125/195) (p=0,096) en varones y de 61,1% (118/193) a 59,6% (115/193) (p=0,755) en mujeres; finalmente, en varones no encontramos casos de hiperglicemia en la medición basal, pero sí uno posintervención; entre mujeres la proporción se mantuvo en 0,5% (1/193) (p=1,00). En la Tabla 2 del material suplementario se muestra la comparación de medianas del perfil lipídico, glucosa sérica, presión arterial y actividad física antes y después de la intervención, estratificado según sexo; en la Tabla 5 se muestra los cambios en el número de componentes de SM entre las mediciones basal y posintervención. Los cambios en la proporción de SM y sus componentes, según estado nutricional, se muestran en la Tabla 4.

**Tabla 5:** Comparación entre el número de componentes de síndrome metabólico en la medición basal y posintervención entre adolescentes, estratificado por sexo.

n= frecuencia, p=proporción, prueba de McNemar-Bowker (prueba a dos colas) en el grupo de varones (p<0,001), mujeres (p<0,001) y total (p<0,001)

Las celdas sombreadas en verde corresponden a participantes que disminuyeron el número de componentes para la medición posintervención, las celdas en gris son aquellos que se mantuvieron con el mismo número de componentes, y las celdas en rojo son aquellos que incrementaron el número de componentes.

Nota para facilitar la lectura de la tabla: Entre 188 adolescentes que tuvieron un componente en la medición basal, 56 dejaron de presentarlo posintervención; entre los 92 varones con un componente, 26 presentaron dicho cambio, y entre las 96 mujeres, 30 también lo tuvieron. Entre 103 estudiantes que tuvieron dos componentes en la medición basal, 55 redujeron a un componente y 13 dejaron de presentarlos.

## DISCUSIÓN

La intervención educativa implementada en dos escuelas de educación secundaria de una región altoandina del Perú generó dos cambios relevantes; primero, un incremento en 12 pp en la proporción de adolescentes sin componentes del SM, y segundo, una reducción significativa en la frecuencia de SM. Luego de culminada la intervención, 11 de 18 adolescentes que inicialmente tenían SM dejaron dicho estado. Este cambio estaría explicado principalmente por el efecto sobre la hipertrigliceridemia, pues 101 de 159 adolescentes pasaron de valores elevados de triglicéridos a normales.

A diferencia de nuestra investigación, la mayoría de las evaluaciones de intervenciones educativas con diseño pre y posintervención en un solo grupo fueron realizados en adolescentes o niños con exceso de peso. Dos estudios fueron realizados en el entorno educativo, uno en 98 niños mexicanos de 6 a 12 años reportó una reducción de 44 a 16% en el SM, además redujo la hipertrigliceridemia (64 a 35%), la HTA (19 a 0%) y el c-HDL≤ 40 mg/dL (60 a 41%) ^17^. El otro estudio fue en Chile y encontró que 28 de 50 estudiantes obesos, que completaron una intervención de 8 meses, lograron reducir el SM de 36 a 18%, este efecto se generó por la disminución en los niveles de glucosa, triglicéridos y c-LDL ^25^. Otros estudios preexperimentales -pero en entornos no escolares- muestran un efecto variado en la reducción del SM. Una intervención en 53 niños y adolescentes de Colombia, participantes de un programa hospitalario para obesidad, redujo el SM de 37 a 32% ^26^. Una intervención comunitaria en 144 niños y adolescentes obesos de los Estados Unidos de Norteamérica encontró una modesta reducción del SM (30,6 a 27,1%) ^27^. Por otro lado, un estudio en 85 niños obesos atendidos en un hospital italiano, encontró una reducción de 17,1 a 4,9% entre las medidas pre y posintervención ^28^.

Una mejor comparación de nuestros resultados se da con un estudio que evaluó el efecto de una intervención educativa sobre el SM en adolescentes provenientes de la población general. El estudio realizado en España en 263 adolescentes de 12 a 16 años, encontró una reducción significativa en el SM (32,2 al 19,7%); en la hipertrigliceridemia (4,7 a 0,8%), y la proporción de afectados por c-HDL≤40 mg/dL (2,8 a 0,7%) ^29^. Si bien observamos resultados consistentes respecto al efecto de estas intervenciones educativas sobre el SM, el tamaño del efecto difiere entre estudios. Dicha variabilidad sería explicada por los diferentes tipos de población diana -sobrepeso u obesidad versus población general-; el entorno de implementación -escuelas, hospitales o comunidad-; la duración y dosificación de las sesiones de la intervención; el tamaño muestral; los criterios de SM -ATP-III, Cook o IDF -, y la prevalencia basal del SM.

Las intervenciones implementadas en el entorno escolar difieren en su duración, en el presente estudio se realizaron nueve sesiones grupales cada 15 días, y cada sesión duró 45 min. Elizondo *et al.* ^(^ ^17^ evaluaron una intervención para niños con exceso de peso mediante trece sesiones individuales (no grupales) cada tres semanas, durante un año escolar; cada sesión duró 30 min. Este estudio encontró que la intervención, además, redujo el IMC e incrementó la actividad física. Campos *et al.* ^29^ diseñaron una intervención para estudiantes de secundaria en general caracterizada por sesiones grupales cada 15 días durante el año escolar, el estudio no precisó número ni duración de sesiones. Dicha intervención también redujo la ingesta de calorías diarias en varones y mujeres. Por otro lado, Bustos *et al*. ^25^ evaluaron una intervención de 12 sesiones nutricionales brindadas en 8 meses para escolares obesos, se realizó una sesión de 40 min por semana; también se realizaron sesiones de actividad física de 50 min, dos veces por semana. Este estudio, además, encontró efecto sobre el IMC y el porcentaje de grasa total en tronco y en las cuatro extremidades.

En el análisis estratificado por sexo, se encontró, tanto en varones como en mujeres, un incremento significativo en la proporción de adolescentes libres de componentes de SM; por otro lado, la reducción en la proporción de SM -en varones y mujeres- no resultó significativa. Se halló de modo consistente una reducción de la hipertrigliceridemia en ambos sexos, esta tendencia no ha sido observada en el estudio de Campos *et al*., donde entre escolares españoles, solo en varones, se encontró una diferencia significativa de la media de triglicéridos antes y después de la intervención ^29^.

La intervención, según el estado nutricional del adolescente, mostró un efecto diferenciado, pues entre obesos no se halló un efecto significativo. Esta menor intensidad de respuesta a las intervenciones en niños y adolescentes obesos ha sido previamente reportada. Este hallazgo podría sugerir la necesidad de intervenciones específicas para reducir el SM en dicho subgrupo ^27^ ^,^ ^29^. Entre estas estrategias específicas se sugieren intervenciones que incorporen programas de ejercicio físico, así como el monitoreo de actividad física en el hogar, dichas adecuaciones deberían ser adicionales al contenido de la intervención ^25^ ^,^ ^29^. El presente estudio y la mayoría de investigaciones han diseñado la intervención para brindarse en un año lectivo ^17^ ^,^ ^25^ ^,^ ^27^ ^,^ ^29^, por lo que para mejorar los resultados en obesos dichas intervenciones deberían evaluarse para más de 12 meses.

Esta intervención produjo una reducción significativa en la proporción de hipertrigliceridemia, tanto en la muestra global como en los estratos definidos por sexo y estado nutricional. Un metaanálisis de ECA para tratar niños y adolescentes con sobrepeso y obesidad encontró que las intervenciones dietéticas redujeron, en promedio, 13,3 mg/dL más las concentraciones de triglicéridos, comparados con el grupo que recibió dieta más programa de ejercicios ^12^. Un estudio preexperimental en niños mexicanos redujo la hipertrigliceridemia (≥110 mg/dL) del 64 al 35% ^17^, mientras que en adolescentes españoles la reducción de participantes con triglicéridos >150 mg/dl fue de 4,7 a 0,8% ^29^. Tanto los triglicéridos como el c-LDL están muy correlacionados con la ingesta de alimentos ^30^, por ello, es plausible un efecto directo de las intervenciones educativas nutricionales sobre los hábitos alimentarios de los adolescentes, que generen no solo una reducción en los niveles de triglicéridos sino también del c-LDL. Si bien se ha descrito que la obesidad y sobrepeso en adolescentes latinos se encuentra asociada con dislipidemias ^31^ ^,^ ^32^, en este estudio la prevalencia de obesidad fue solo del 8%, mientras que la de hipertrigliceridemia fue del 41,0%; por ello, es poco probable que el efecto sobre los triglicéridos sea explicada por la reducción del exceso de peso.

La reducción de 3,8 pp en la proporción de estudiantes con niveles de c-HDL bajo no fue significativa, este hallazgo difiere de un estudio -no restringido a adolescentes con exceso de peso- en escolares españoles de 12 a 16 años en el cual se encontró un incremento significativo de la media del nivel de c-HDL entre las mediciones pre y posintervención ^29^. El efecto favorable de intervenciones educativas sobre el incremento de los niveles de c-HDL también ha sido encontrado en estudios preexperimentales en adolescentes con exceso de peso ^17^ ^,^ ^27^ ^,^ ^28^. Al igual que el presente estudio, Bustos *et al*. reportaron una disminución en la media de los niveles de c-HDL en niños y adolescentes chilenos ^25^. Nuestro hallazgo podría deberse a que 35 estudiantes con valores de c-HDL>40 mg/dL en la medición basal pasaron a niveles ≤40 md/dL posintervención, este fenómeno correspondería a un potencial sesgo de maduración, pues se ha visto en adolescentes de 10 a 17 años del Brasil que los niveles del c-HDL tienden a reducir aproximándose a una media de 40 mg/dL a mayor edad ^33^.

Las limitaciones del estudio están vinculadas a la ausencia de grupo control; sin embargo, implementamos la intervención durante 6 meses y las mediciones basal y posintervención fueron realizadas en dicho plazo, así se redujo el efecto de maduración biológica ^34^ y la pérdida de participantes. En ambas mediciones no se realizaron determinaciones repetidas para los niveles de glucosa, triglicéridos y c-HDL, lo cual podría afectar la precisión de dichas mediciones y con ello generar un potencial sesgo de regresión a la media, que afecte la estimación del efecto de la intervención. Para reducir dicho riesgo las determinaciones se hicieron en ayunas, con un adecuado tratamiento preanalítico de la muestra y empleando procedimientos de laboratorio estandarizados y calibrados, reduciendo de esta manera el sesgo de instrumentación. La intervención se llevó a cabo en dos escuelas, una exclusiva para mujeres y otra para varones, por ende, algunas diferencias entre ambos entornos, que no fueron medidas en el estudio, podrían confundir el efecto de la intervención. No se evaluaron desenlaces intermedios tales como cambios en el consumo de alimentos o el nivel de conocimientos sobre alimentación saludable. Finalmente, no se pudo excluir potenciales casos de dislipidemias primarias, lo cual, según su prevalencia, podrían generar un sesgo de selección.

En conclusión, la intervención educativa en nutrición y estilos de vida saludables implementada en dos escuelas de un área andina del Perú generó un incremento en el número de adolescentes libres de componentes de SM, así como una reducción significativa en la proporción de adolescentes con SM, este último efecto es explicado, principalmente, por el cambio producido en los niveles de triglicéridos. Se recomienda evaluar el efecto de la intervención en adolescentes en escuelas de la costa y selva, estas deberían adaptarse a la disponibilidad de alimentos de la región, así como el contexto cultural.
